# The distinct role of strand-specific miR-514b-3p and miR-514b-5p in colorectal cancer metastasis

**DOI:** 10.1038/s41419-018-0732-5

**Published:** 2018-06-07

**Authors:** Lin-Lin Ren, Ting-Ting Yan, Chao-Qin Shen, Jia-Yin Tang, Xuan Kong, Ying-Chao Wang, Jinxian Chen, Qiang Liu, Jie He, Ming Zhong, Hao-Yan Chen, Jie Hong, Jing-Yuan Fang

**Affiliations:** 1 State Key Laboratory for Oncogenes and Related Genes; Division of Gastroenterology and Hepatology; Key Laboratory of Gastroenterology and Hepatology, Ministry of Health; Renji Hospital, School of Medicine, Shanghai JiaoTong University, Shanghai Institute of Digestive Disease, 145 Middle Shandong Road, 200001 Shanghai, China; 2 Department of Gastroenterology, The Affiliated Hospital of Qingdao University, Shandong Sheng, China; 3 Department of Surgery, Renji Hospital, Shanghai Jiao-Tong University School of Medicine, Shanghai, China; 4 Department of Pathology, Renji Hospital, Shanghai Jiao-Tong University School of Medicine, Shanghai, China; 5 Department of Gastroenterology & Guangzhou Key Laboratory of Digestive Disease, Guangzhou Digestive Disease Center, Guangzhou First People’s Hospital, Guangzhou Medical University, Guangzhou, China

## Abstract

The abnormal expression of microRNAs (miRNAs) in colorectal cancer (CRC) progression has been widely investigated. It was reported that the same hairpin RNA structure could generate mature products from each strand, termed 5p and 3p, which binds different target mRNAs. Here, we explored the expression, functions, and mechanisms of miR-514b-3p and miR-514b-5p in CRC cells and tissues. We found that miR-514b-3p was significantly down-regulated in CRC samples, and the ratio of miR-514b-3p/miR-514b-5p increased from advanced CRC, early CRC to matched normal colorectal tissues. Follow-up functional experiments illustrated that miR-514b-3p and miR-514b-5p had distinct effects through interacting with different target genes: MiR-514b-3p reduced CRC cell migration, invasion and drug resistance through increasing epithelial marker and decreasing mesenchymal marker expressions, conversely, miR-514b-5p exerted its pro-metastatic properties in CRC by promoting EMT progression. MiR-514b-3p overexpressing CRC cells developed tumors more slowly in mice compared with control cells, however, miR-514b-5p accelerated tumor metastasis. Overall, our data indicated that though miR-514b-3p and miR-514b-5p were transcribed from the same RNA hairpin, each microRNA has distinct effect on CRC metastasis.

## Introduction

Colorectal cancer (CRC) is the third most common and the fourth leading cause of cancer related mortality worldwide^1^. Due to the change in diet, the incidence of CRC in Asian countries increased remarkably during the past decades^2^. Although the surgical resection and postoperative chemotherapy help to get a better outcome for early-stage CRC, the clinical output for advanced CRC is still unsatisfactory, mainly due to metastasis^2^. Therefore, it is of critical importance to elucidate the underlying mechanism involved in metastasis process and identify biomarkers for CRC.

Epithelial mesenchymal transition (EMT) plays a pivotal role in cancer invasion and metastasis^3^. Within the normal intestinal epithelium, cells interact with each other to form tissue integrity. During the EMT process, the cell–cell junction disassembled and cancer cells lose their apico-basal polarity and gain the ability to migrate from the primary tumor into the surrounding tissue, which is the first step toward metastasis^4,5^. Besides, cells involved in EMT have many traits with stem cells in common, such as increased drug resistance, causing systemic therapy more complicated for metastatic disease^6,7^.

MicroRNAs (miRNAs) are small (20–25 nucleotides) non-coding RNAs. They regulate gene expression post-transcriptionally through inhibiting the translation or reducing the stability of messenger RNAs (mRNAs), directly binding to 3′ untranslated regions (3′UTRs) of target mRNA in a sequence-specific way^8^. Emerging evidence has demonstrated that miRNAs dysfunction is involved in various physiological or pathological process, including cell proliferation, apoptosis, drug resistance, and tumor metastasis^9–13^. In the miRNA biogenesis pathway, the precursor miRNA (pre-miRNA) are transcripted by RNA polymerase II (Pol II) from the DNA template and spliced into the primary miRNA (pri-miRNA) via Drosha in the nucleus. Then the pri-miRNA are transported to cytoplasm by the Exportin-5 complex and then processed to the mature miRNA via the Dicer enzyme^14^. Since the pri-miRNA has two arms, namely 3p and 5p arm, mature miRNAs excised from different arms of the same primary transcript have different sequences and therefore function diversely^15^.

It was reported that miR-514a could function as a tumor suppressor gene in testicular germ cell tumor, melanoma and renal cell carcinoma. However, there is no study investigating the function of miR-514b in CRC^16–18^. Here, we found that miR-514b-3p and miR-514b-5p displayed opposite functions in CRC development. The ratio of miR-514b-3p/5p is higher in normal tissue compared with paired CRC tissue and decrease with the tumor progression. These results indicate a potential role for the miR-514 family in CRC evolution and metastasis.

## Materials and methods

### Clinical specimen collection

In all, 62 freshly-frozen CRC tissues and adjacent non-cancerous tissues in Renji cohort 1 were obtained from patients who underwent surgery in Renji Hospital from 2010 to 2015. Similarly, we collected paraffin-fixed CRC tissues and normal colorectal tissues from 40 patients with CRC between 2014 and 2016 at Shanghai Renji Hospital (Renji cohort 2). The clinicopathological information was provided by Pathology Department of Renji Hospital and the clinicopathological traits involved gender, age, tumor size, clinical stage (AJCC), T classification, lymph node metastasis, distant metastasis, vascular invasion, and histological differentiation. None of patients had received chemotherapy or radiotherapy before surgery. Written informed consent was obtained from all patients involved in this study and ethical consent was granted from Shanghai Jiao Tong University School of Medicine, Renji Hospital.

### Bioinformatics analysis

Gene set enrichment analysis (GSEA) was performed using the GO gene sets database (c2.all.v4.0.symbols.gmt) from the Molecular Signatures Database–MsigDB. Enrichment results were generated from 1000 random permutations and significance was estimated by comparing the enrichment score. FDR, 0.25 was considered to be a cut-off to identify biologically relevant genes.

### Cell cultures and treatments

CRC cell lines LOVO, HCT116, SW620, SW480, SW1116, HT29, and Caco2 were purchased from ATCC (American type culture collection). All CRC cells were cultured in medium with 10% FBS (fetal bovine serum) at 37 ℃ with an atmosphere of 5% CO_2_.

The miRNA mimics or miRNA inhibitors were transfected into CRC cell lines using DharmaFECT 1 transfection reagent (Thermo Scientific, USA) or Lipofectamine 3000 (Invitrogen, USA) according to the manufacturer’s instruction. All the miRNA mimics and miRNA inhibitors were synthesized and purchased from Genepharm Technologies (China). The sequences of miRNA mimics involved in the study were listed as follows: miR-514b-3p mimics: AUUGACACCUCUGUGAGUGGA; miR-514b-5p mimics: UUCUCAAGAGGGAGGCAAUCAU; miR-514b-3p inhibitor: UCCACUCACAGAGGUGUCAAU; miR-514b-5p inhibitor: AUGAUUGCCUCCCUCUUGAGAA.

### Total RNA extraction and real-time PCR

Total RNA of CRC cell lines, CRC tissues and adjacent non-cancerous tissues was isolated using Trizol reagent (Takara, Japan). First-strand cDNA was synthesized using the All-in-One miRNA First-Strand cDNA Synthesis Kit (Genecopeia, USA) and real-time PCR was conducted using ABI (Thermo Fisher Scientific, USA) in the StepOne real-time PCR system. The relative mRNA expression levels were quantified by the 2^−ΔΔCt^ method. U6 and GAPDH were used as internal controls. The gene-specific primers used in this study were listed as follows: CDH1-F: AAAGGCCCATTTCCTAAAAACCT; CDH1-R: TGCGTTCTCTATCCAGAGGCT; CLDN1-F: CCTCCTGGGAGTGATAGCAAT; CLDN1-R: GGCAACTAAAATAGCCAGACCT; FZD4-F: CCTCGGCTACAACGTGACC; FZD4-R: TGCACATTGGCACATAAACAGA; NTN1-F: GGGTGCCCTTCCAGTTCTAC; NTN1-R: GCGAGTTGTCGAAGTCGTG; GAPDH-F: GCATTGCCCTCAACGACCAC; GAPDH-R: CCACCACCCTGTTGCTGTAG; Vimentin-F: TTGACCTTGAACGCAAAGTG; Vimentin-R: TTTGGACATGCTGTTCCTGA; Fibronectin-F: GCCTTCAAGTTCCCCTGTTAC; Fibronectin-R: GACTCTCTCCGCTTGGATTCT;

### Protein extraction and western blot

Protein extraction buffer (Beyotime, China), which contained a protease inhibitor mixture (protease inhibitors, phosphatase inhibitors, PMSF) was used to extract total protein. The standard curve of protein concentration was plotted using BCA Protein Assay Kit (Pierce Biotechnology). Proteins were separated by SDS-polyacrylamide gels and transferred to PVDF membranes. The membranes were incubated with second antibody accordingly overnight at 4℃ after blocked with 5% BSA (Bull Serum Albumin). The next day the membranes were incubated with species-specific secondary antibodies and visualized by the ECL detection system. Antibodies used in this study were listed as follows: Fibronectin (Abcam, UK), Vimentin (Abcam, UK), CLND1 (CST, USA), CDH1 (CST, UK) and GAPDH (KangChen, China). GAPDH was used as an internal control. The dilution of antibodies was 1:1,000.

### Transwell invasion assay

LOVO and HCT116 cells were planted into 6-well plates and transfected with miRNA mimics or inhibitor accordingly. The cells were collected 24 h later and suspended in serum-free culture medium. 2 × 10^5^ cells with 200 µl serum-free culture medium were seeded into the upper chambers which were coated with fresh matrigel and 600 µl culture medium with 20% FBS was added into the lower chambers. 48 h later, the matrigel and the cells on the upper chambers were removed gently and the cells on the lower chambers were fixed with 4% paraformaldehyde and stained with 0.1% crystal violet. The invasion cells were counted under microscope.

### Scratch assay

CRC cell lines were planted into 6-well plates and transfected with miRNA mimics accordingly. Scratch wounds were made by scraping the monolayer of cells when the cells were grown to almost 90% density. The wounded areas were observed at the time point of 0 h and 24 h and measured under a light microscope.

### ELISA

The expression of MMP2 and MMP9 in the cell supernatants was quantified using a human MMP2 and MMP9 ELISA kit (RayBiotech, USA) according to the manufacturer’s guide.

### Cell proliferation assays

HCT116 and LOVO cells were planted into 96-well plates and transfected with miRNA mimics the next day. Cells were treated with Cell Counting Kit-8 (CCK8, Dojindo, Japan) for 2 h at 37 ℃ at the time point of 24 h, 48 h, 72 h, 96 h, and 120 h after seeding. The cell proliferation curves were plotted using the absorbance, which was measured with wavelength 450 nm (450 OD). Each group was measured in 6 replicate wells and all the experiments were repeated at least 3 times.

### Cell cycle analysis

CRC cells were seeded into 6-well plates and transfected with miRNA mimics accordingly. Cells were harvested 48 h after transfection and fixed with 70% pre-cooling ethanol at -20℃ overnight. The supernatant was discarded after centrifugation and the cells were washed with PBS for 2 times the next day. Then the cells were stained with 500 μL PI/RNase staining buffer (BD Pharmingen, USA) for 15 min in the dark. Finally, the samples were analyzed using a fluorescence-activated cell-sorting flow cytometer (BD Biosciences, USA).

### In situ hybridization

The in situ detection of miR-514b-3p and miR-514b-5p was performed on 6-μm formalin-fixed, paraffin-embedded (FFPE) sections using DIG-labeled miRCURYTM Detection probe (Exiqon, Denmark). Briefly, the slides were hybridized with a probe (LNA-modified and DIG-labeled oligonucleotide; Exiqon) complementary to miR-514b-3p/miR-514b-5p and after incubation with anti–DIG-AP Fab fragments conjugated to alkaline phosphatase. The hybridized probes were then detected by applying nitroblue tetrazolium/5-bromo-4-chloro-3-indolyl phosphate color substrate (Roche) to the slides. Slides were counterstained with VECTOR® nuclear fast red counterstain (VECTOR LABOTATORIES) and analyzed with a Nikon 80i microscope and Nikon NIS-Elements F 2.3 software (Nikon). The sequences of miRNA probe involved in the study were listed as follows: miR-514b-3p probe: /5DigN/TCCACTCACAGAGGTGTCAAT/3Dig_N/; miR-514b-5p probe: /5DigN/TCTACTCACAGAAGTGTCAAT/3Dig_N/.

The slides were examined independently by two investigators, who were blinded to the clinical and pathological data. MiR-514b-3p and miR-514b-5p expression were quantified using a visual grading system based on the extent of staining (percentage of positive tumor cells) and the intensity of staining (graded on a scale of 1–3: 1, weak staining; 2, moderate staining; 3, strong staining). For further analysis, the product (the corresponding score) of the extent and intensity grades was used to define the expression levels of the miRNA.

### Nude mice metastatic model

HCT116 cells were infected with lenti-514b-3p and lenti-514b-5p to construct HCT116-514b-3p and HCT116-514b-5p stable expressing cell lines. Cells were collected and suspended in PBS. Male BALB/c nude mice aged 4 weeks old were purchased from Animal Center of Tongji University and maintained under SPF conditions. A total of 1 × 10^7^ suspended cells were injected into the dorsal right flank of mice. Survival times of the mice were recorded. All the mice were euthanized 12 weeks after inoculation and lungs were obtained for fixation and HE staining. The animal experiments were approved by the Institutional Animal Care and Use Committee.

### Chemotherapy resistance assays

CRC cell lines were planted into 96-well plates and transfected with miRNA mimics the next day. 6 h after transfection, the cells were treated with Irinotecan or Cisplatin with different concentrations. After 48 h of incubation, the cells were treated with Cell Counting Kit-8 (CCK8, Dojindo, Japan) for 2 h at 37 ℃. The absorbance was detected at wave length 450 nm using a microplate reader. Each group was down in six replicate wells and all the experiments were repeated at least 3 times.

### In vivo chemotherapy resistance assays

HCT116 cells were infected with lenti-514b-3p, lenti-514b-5p and lenti-control virus to construct HCT116-514b-3p and HCT116-514b-5p stable expressing cell lines. Cells were collected and suspended in PBS. Male BALB/c nude mice aged 4 weeks old were purchased from Animal Center of Tongji University and maintained under SPF conditions. A total of 5 × 10^6^ suspended cells were injected into the dorsal right flank of mice. When the volume of tumors reached 200 mm^3^, the mice were treated with 40 mg/kg Irinotecan, 2.5 mg/kg Cisplatin or PBS. The tumor sizes were measured every 3 days. The tumor volumes were calculated using the formula: *V* = (length x width^2^)/2, (*V*, volume). The animal experiments were approved by the Institutional Animal Care and Use Committee.

### Luciferase reporter assays

Luciferase reporter plasmids containing CDH1, CLDN1, FZD4, or NTN1 3′ UTR miRNA target sequences (pGL3-CDH1, pGL3-CLDN1, pGL3-FZD4, pGL3-NTN1 and their respective miRNA target mutants) were generated. CRC cells were seeded into 24-well plates the day before transfection. For each well, 0.5 μg pGL3 together with 0.05 μg Renilla vector (Generay, China) were cotransfected with 3 μL miRNAs using Lipofectamine 3000 (Invitrogen, USA). 48 h later, cells were lysed with 500 μL passive lysis buffer and 10 μL supernatant was used for further detection of luciferase activity using the dual luciferase reporter assay system (Promega, USA) according to the manufacturer’s instruction. Renilla luciferase activity was used to normalize firefly luciferase activity.

### Statistical analysis

All statistical analyses were carried out using the GraphPad Prism 5 and SPSS 19.0 software. Association between miR-514b-3p and miR-514b-5p expression and clinicopathologic parameters of CRC patients was analyzed using chi-square test. Overall survival of metastatic animal model was analyzed using the log-rank test and was plotted using Kaplan–Meier survival curve. All the experiments were repeated at least three times and the data were presented in the form of means ± SEM. *p* < 0.05 was considered to be significant.

## Results

### Different profiles of miR-514b-3p and miR-514b-5p in CRC tissues

**Fig. 1:**
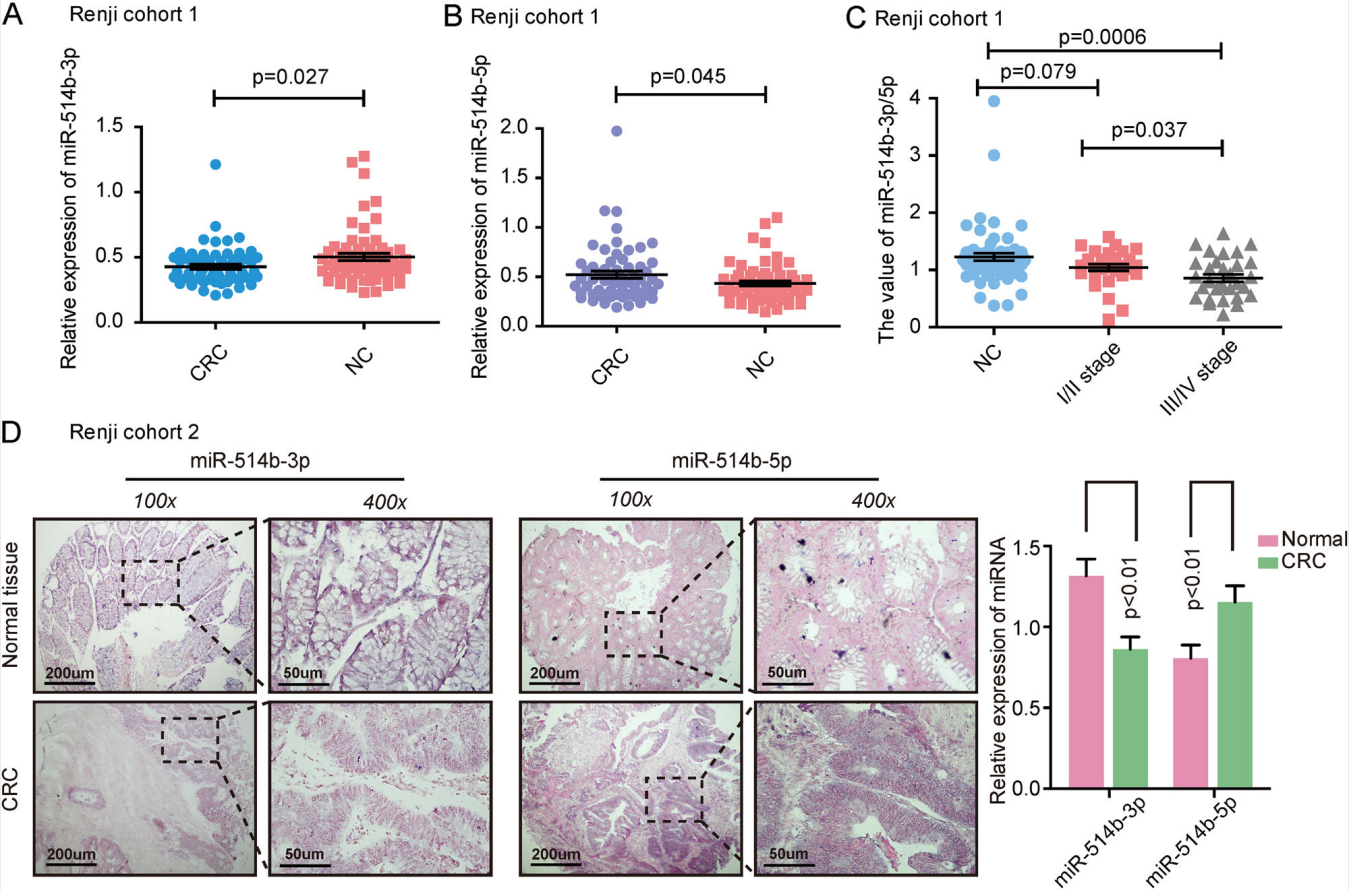
The expression of miR-514b-3p and miR-514b-5p in CRC tissues. **a**, **b** The expression of miR-514b-3p and miR-514b-5p was measured by real-time PCR in CRC tissues and matched non-tumor tissues in Renji cohort 1 (*n* = 62). Error bars represented SEM. **c** The ratio of miR-514b-3p/miR-514b-5p was analyzed in different stages of CRC progression. **d** The expression of miR-514b-3p and miR-514b-5p was measured by ISH (in situ hybridization) and the summarized data was presented in CRC tissues and matched non-tumor tissues in Renji cohort 2 (*n* = 40), the purple staining in the image indicates the specific microRNA ISH signal and red indicates the nuclear counter stain

The miR-514b-3p and miR-514b-5p expression were analyzed in Renji cohort 1, consisting of 62 pairs of CRC tissues and adjacent normal tissues. The results showed that miR-514b-3p expression was significantly down-regulated in CRC samples (Fig. 1a, *p* < 0.05), while miR-514b-5p was significantly up-regulated in CRC samples compared with matched normal tissues (Fig. 1b, *p* *<* *0.05*). Additionally, the In situ hybridization (ISH) data displayed the similar results in Renji cohort 2 patients (Fig. 1d). We further determined the ratio of miR-514b-3p/miR-514b-5p in different CRC stages in Renji cohort 1. The data indicated that the ratio of miR-514b-3p/miR-514b-5p was gradually decreased from normal colorectal tissue to stage I-II and stage III-IV of CRC tissues (Fig. 1c). We further analyzed the association between miR-514b-3p/miR-514b-5p value and clinicopathological features of CRC in Renji cohort 1. The data revealed that high miR-514b-3p/miR-514b-5p level was negatively correlated with tumor stage, histological differentiation and lymphnode invasion (Supplementary Table 1). Overall, these data indicated that miR-514b-3p and miR-514b-5p were highly associated with CRC development.

### MiR-514b-3p may inhibit cell invasion and metastasis while miR-514b-5p may promote these cell processes in CRC

**Fig. 2:**
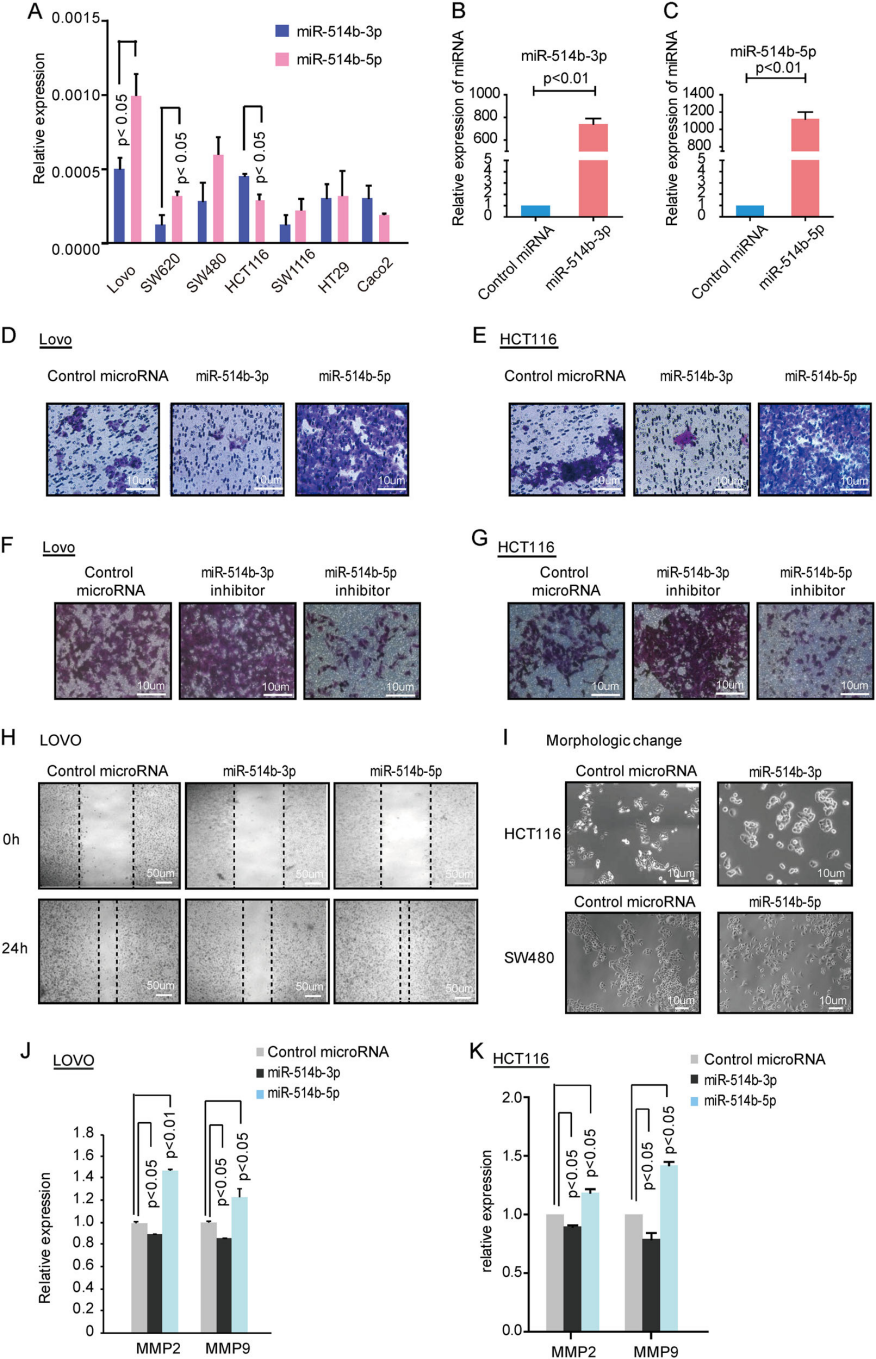
The effects of miR-514b-3p and miR-514b-5p on CRC invasion and metastasis in vitro. **a** The relative expression of miR-514b-3p and miR-514b-5p was quantified by real-time PCR in CRC cell lines. **b**, **c** The overexpression efficiency was confirmed in HCT116 and LOVO cells by real-time PCR. **d**, **e** Transwell Matrigel invasion assays were performed in HCT116 and LOVO cells after transfection of miR-514b-3p or miR-514b-5p mimics. **f**, **g** Transwell invasion assays were performed in HCT116 and LOVO cells after transfection of miR-514b-3p or miR-514b-5p inhibitors. **h**. The scratch assays were conducted in LOVO cells after control, miR-514b-3p and miR-514b-5p mimics transfection. **i**. The morphologic changes were observed under a light microscope in HCT116 and SW480 cells transfected with miR-514b-3p or miR-514b-5p mimics. **j**, **k** The effects of miR-514b-3p and miR-514b-5p on MMP2 and MMP9 expression were evaluated using ELISA in LOVO and HCT116 cells. All the experiments above were done for three replicates and repeated at least three times. Error bars represented SEM

To gain a comprehensive understanding of the role of miR-514b-3p and miR-514b-5p in CRC tumorigenesis, two CRC cell line HCT116 and LOVO with low expression of miR-514b-5p or miR-514b-3p were transfected with miR-514b-3p, miR-514b-5p or control miRNA (Fig. 2a). The overexpression efficiency was confirmed by qRT-PCR (Fig. 2b, c). We first explored the miRNA-mediated cell proliferation and cell cycle arrest in HCT116 and LOVO cell lines using CCK8 assays and flow cytometry, respectively. The data showed that miR-514b-3p and miR-514b-5p had no significant difference in cell proliferation or cell cycle arrest in the two cells compared with negative control (Supplementary Fig. 1A-1C).

Next, we analyzed the potential biological effect of miR-514b-3p and miR-514b-5p on cell migration and invasion in HCT116 and LOVO cells. Overexpression of miR-514b-3p significantly suppressed cell motility and invasiveness in both HCT116 and LOVO cells, while those cells transfected with miR-514b-5p mimics showed increased invasion ability, compared with control miRNA (Fig. 2d, e). The opposite effects were observed when cells were treated with miRNA inhibitors (Fig. 2f, g). The wound healing assay and morphological changes in cells transfected with miR-514b-3p and miR-514b-5p, further confirmed our results (Fig. 2h, i). Matrix metalloproteinase 2 (MMP2) and MMP9 possess ability to hydrolyze components of the basement membrane, and thus could promote tumor metastasis. To better understand the function of miR-514b-3p/5p in CRC metastasis, MMP2 and MMP9 were detected in cell culture supernatant by ELISA. We observed that restoration of miR-514b-3p significantly reduced MMP2 and MMP9 expression, whereas miR-514b-5p increased MMP2 and MMP9 secretion in LOVO and HCT116 cells (Fig. 2j, k).

**Fig. 3:**
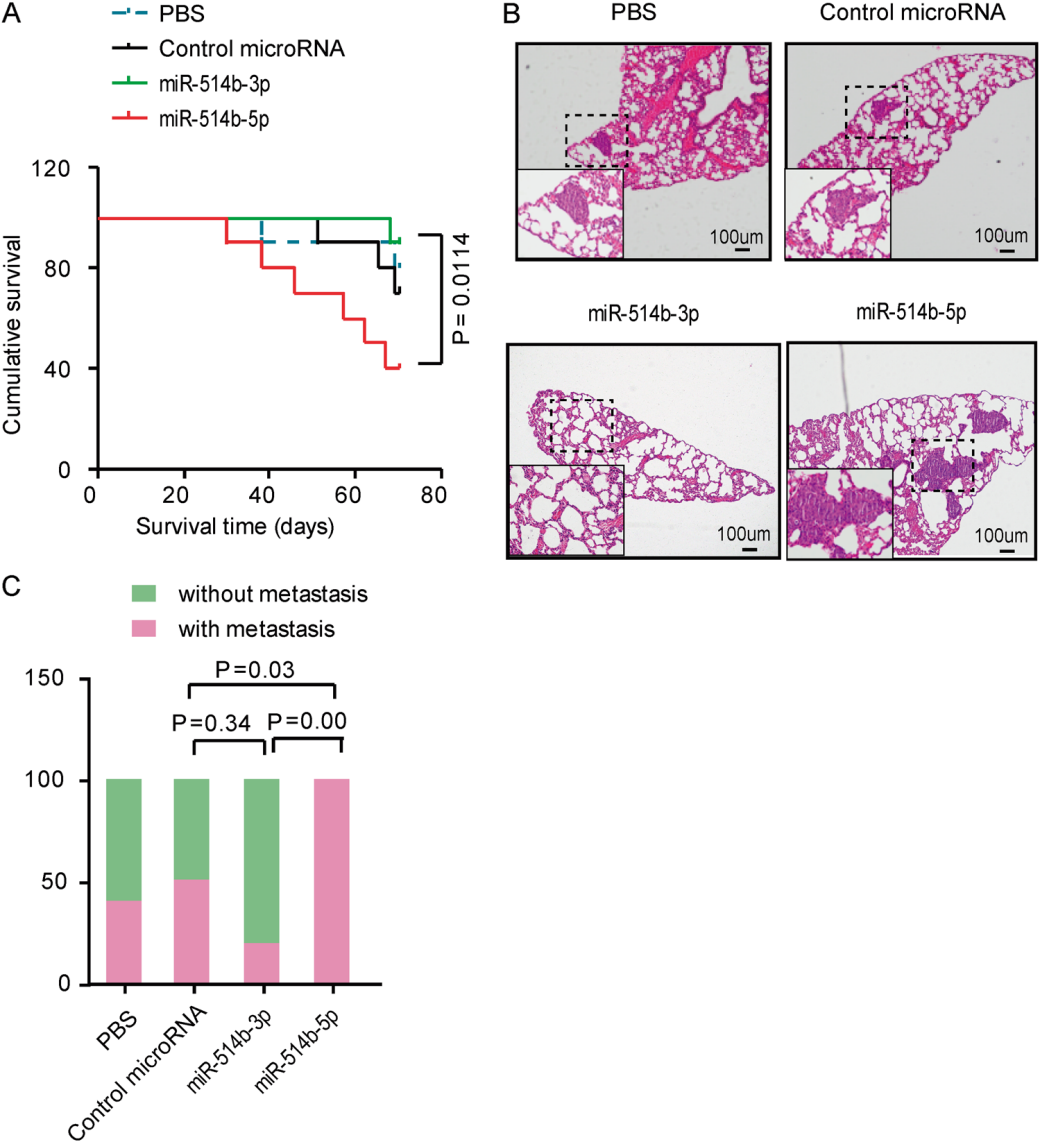
The effects of miR-514b-3p and miR-514b-5p on CRC metastasis in vivo. **a** Survival time was analyzed in nude mice bearing HCT116-control miRNA cells, HCT116-miR-514b-3p, HCT116-miR-514b-5p stable expressing cells or PBS. **b** Representative hematoxylin-eosin staining images of lung metastatic loci in PBS group, control-miRNA group, miR-514b-3p overexpression group and miR-514b-5p overexpression group. **c** The ratio of mice with metastatic loci was analyzed in each group

To further identify the involvement of miR-514b-3p and miR-514b-5p in vivo, we established a CRC metastasis model in nude mice. The HCT116-control miRNA cells, HCT116-miR-514b-3p and HCT116-miR-514b-5p stable expressing cells were subcutaneously injected through the right flank of nude mice, respectively. The mice inoculated with HCT116-miR-514b-3p stable expressing cells had a longer overall survival time compared with those injected with control cells (Fig. 3a). Whereas mice bearing HCT116-miR-514b-5p expressing stable cells had a significant poor survival (Fig. 3a). Hematoxylin-eosin staining showed that less metastatic CRC loci were observed in miR-514b-3p group compared to control group, while all of the mice injected with HCT116-miR-514b-5p stable expressing cells displayed lung metastases (Fig. 3b-c). Altogether, these data indicate that miR-514b-3p and miR-514b-5p may display converse function in CRC invasion and metastasis.

### Distinct roles of miR-514b-3p and miR-514b-5p in chemoresistance

**Fig. 4:**
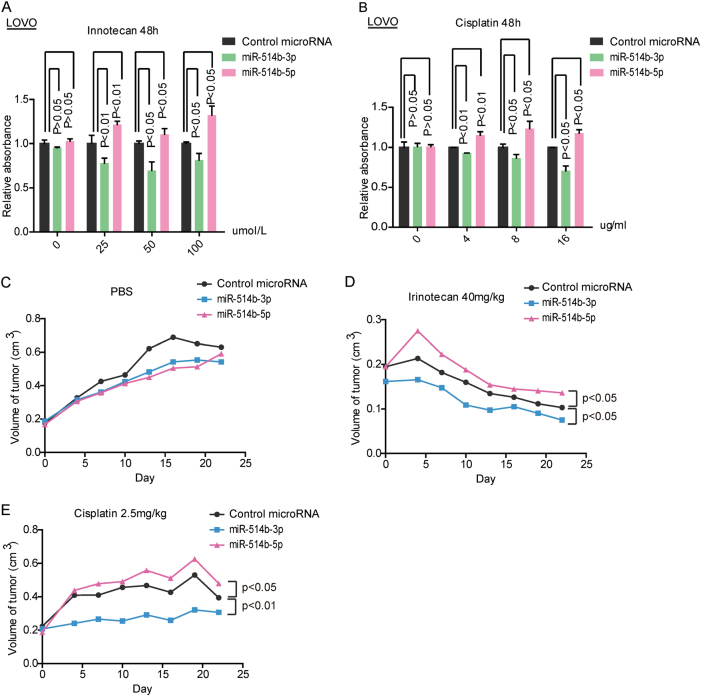
The effects of miR-514b-3p and miR-514b-5p on drug resistance. **a**, **b** Chemotherapy resistance assays were conducted to assess the potential role of miR-514b-3p and miR-514b-5p in the chemoresistance of Cisplatin and Irinotecan. **c**–**e** The tumor volumes were measured in mice with different treatments in the xenograft mouse model at the time point of 0, 5, 10, 15, 20, and 25 days

Since metastasis and chemoresistance in cancer are linked phenomenon^19,20^, we further assessed the potential role of miR-514b-3p and miR-514b-5p in the chemoresistance of Cisplatin and Irinotecan. The data revealed that cells with miR-514b-5p overexpression got an increased viability compared to control miRNA, and there is no significant difference in the absence of Cisplatin or Irinotecan. However, overexpression of miR-514b-3p accelerated cell death in cells treated with Cisplatin or Irinotecan compared to the control cells (Fig. 4a, b).

To better verify the exact role of miR-514b-3p and miR-514b-5p in chemoresistance in vivo, we established CRC xenograft mouse model using HCT116 cell lines. Delivery of miR-514b-3p or miR-514b-5p lentivirus had no effect on the size of tumor in the absence of Cisplatin or Irinotecan (Fig. 4c). However, when treated with Cisplatin (2.5 mg/kg) or Irinotecan (40 mg/kg), mice bearing HCT116-miR-514b-3p cells showed decreased size of tumors, whereas mice inoculated with HCT116-miR-514b-5p cells displayed an increase in tumor growth compared to control group (Fig. 4d, e). Collectively, these findings suggested that miR-514b-3p and miR-514b-5p displayed a possible role in mediating chemoresistance to Cisplatin and Irinotecan.

### MiR-514b-3p and miR-514b-5p mediate CRC cell invasion via regulation of different targets

**Fig. 5:**
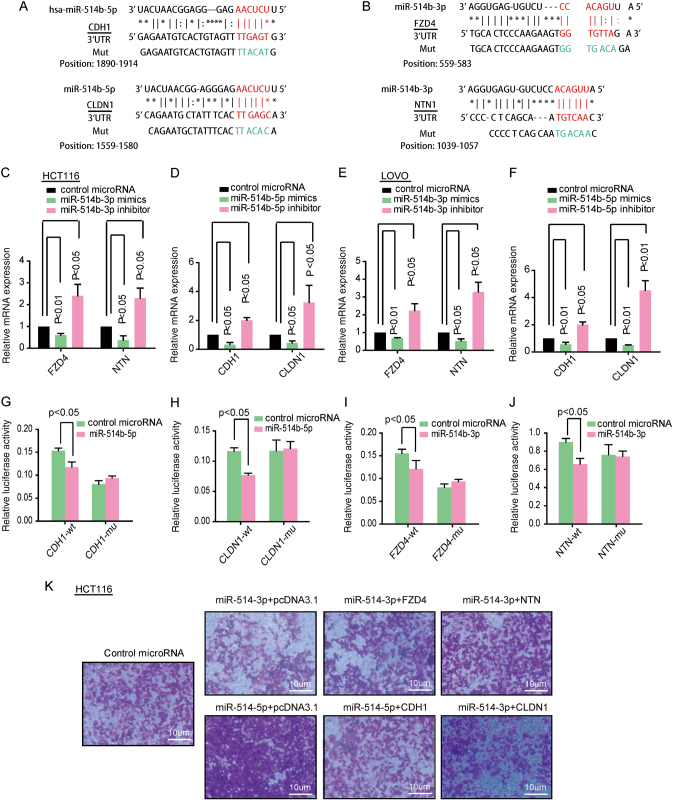
The target genes of miR-514b-3p and miR-514b-5p. **a** Schematic diagram of the predicted binding sites for miR-514b-5p in 3′UTR of CDH1 and CLDN1. **b** Schematic diagram of the predicted binding sites for miR-514b-3p in 3′UTR of FZD4 and NTN1. **c**–**f** The mRNA expression of corresponding target genes was tested by real-time PCR in HCT116 and LOVO cells transfected with miR-514b-3p and miR-514b-5p mimics or inhibitors, respectively. **g**–**j** Luciferase activities were detected in HCT116 and LOVO cells co-transfected with corresponding miRNA mimics and the indicated target gene wild-type reporter plasmids or mutant-type reporter plasmids. The experiments were done for four replicates and repeated at least three times. Error bars represented SEM. **k** The transwell invasion assays were performed in HCT116 after co-transfection with miR-514b-3p or miR-514b-5p mimics and their target genes overexpression plasmid. The experiments were done for three replicates and repeated at least three times

To determine the underlying mechanism of miR-514b-3p and miR-514b-5p, which is involved in CRC invasion and metastasis. The miRBase, microRNA.org and TargetScan website were used to predict target genes of miR-514b-3p and miR-514b-5p. Target prediction programs indicated that there were potential specific targets for miR-514b-3p in the seed regions of oncogenes FZD4 and NTN1, and for miR-514b-5p within the 3′UTR region of CDH1 and CLDN1 (Fig. 5a, b).

To verify the prediction, qPCR was used to test the target mRNA expression in HCT116 and LOVO cells transfected with miR-514b-3p and miR-514b-5p mimics or inhibitors, respectively. MiR-514b-3p and miR-514b-5p overexpression significantly decreased their corresponding target genes expression, and the inhibitors showed opposite effects (Fig. 5c–f). To explore whether the effect was mediated by directly binding of the miRNAs to the seed regions of their target genes, we constructed the recombined luciferase reporter plasmid, containing the predicted micoRNA binding sites of target genes. Luciferase assay data revealed that when co-transfected with wild-type reporter plasmids (CDH1 and CLDN1) and miR-514b-5p mimics, the luciferase activity was significantly decreased, whereas the mutant-type reporter plasmids made no difference (Fig. 5g, h). Also, we find that miR-514b-3p mimics significantly decreased the luciferase activity of wild-type 3′UTR (FZD4 and NTN1), but did not affect the activity of mutant-type reporter plasmids (Fig. 5i, j). These data indicated that miR-514b-5p promoted CRC progression by targeting CDH1 and CLDN1, and miR-514b-3p inhibited CRC development through direct targeting FZD-4 and NTN1.

To further confirm our suggestion, we co-transfected HCT116 cells with miR-514b-3p mimics and their target gene. As shown in Fig. 5k, overexpression of FZD4 and NTN1 partially blocked miR-514b-3p induced anti-invasion. The same phenomenon was observed when cells were co-transfected with miR-514b-5p and its target genes’ overexpression plasmids. Collectively, the data revealed that miR-514b-3p and miR-514b-5p regulated CRC cell invasion through downregulation of different target genes.

### The functional role of miR-514b-3p and miR-514b-5p in the EMT progression of CRC

**Fig. 6:**
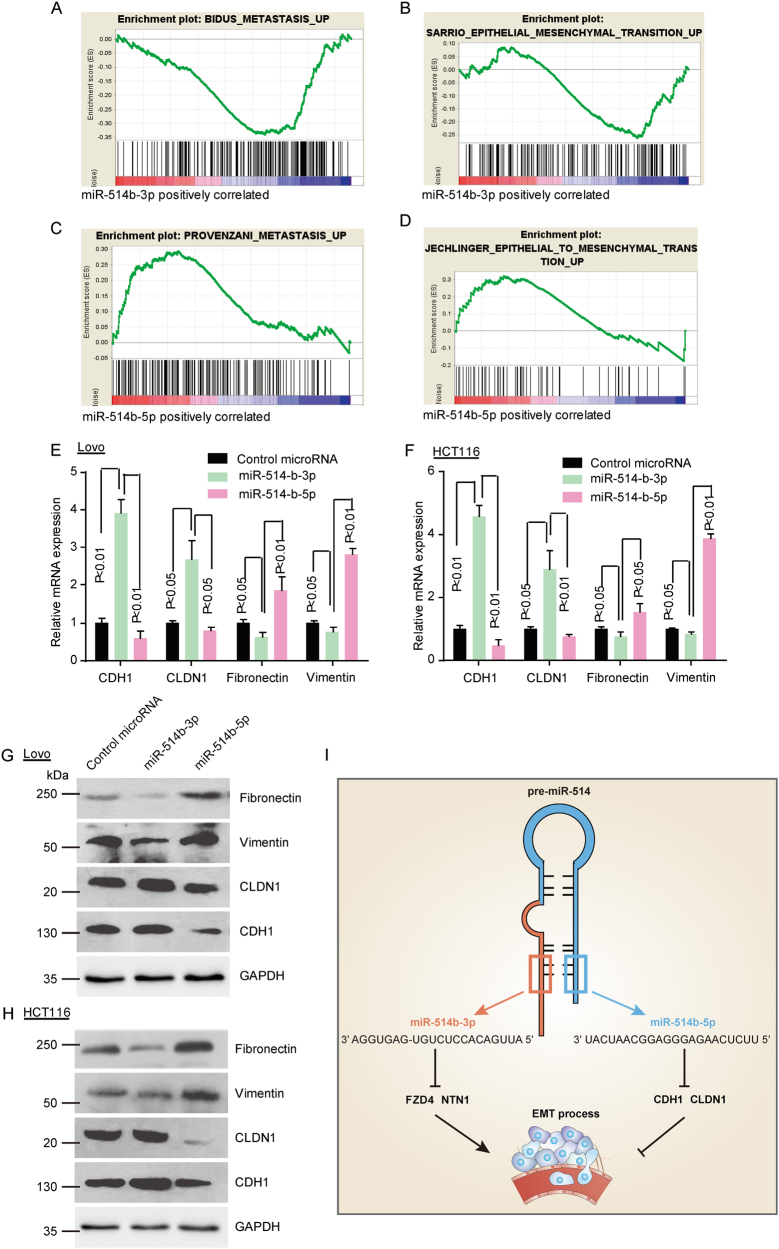
The functional role of miR-514b-3p and miR-514b-5p in the EMT progression of CRC cell lines. **a**–**d** GSEA was performed to identify the difference of gene profiles in control-miRNA group, miR-514b-3p overexpression group and miR-514b-5p overexpression group. **e**, **f** Real-time PCR was performed to measure the mRNA expression of EMT markers (E-cadherin, CLDN-1, fibronectin-1 and vimentin) in LOVO and HCT116 after transfection of miR-514b-3p or miR-514b-5p mimics. Error bars represented SEM. **g**, **h** Western-blot was conducted to evaluate the protein expression of EMT markers (E-cadherin, CLDN-1, fibronectin-1, and vimentin) in LOVO and HCT116 after transfection of miR-514b-3p or miR-514b-5p mimics. GAPDH was used as an internal control. **i** Schematic representation for the mechanism of miR-514 regulates EMT in human CRC progression

As EMT is well-known to be involved in invasion and metastasis in cancer, we are supposed to investigate whether miR-514b-3p and miR-514b-5p take part in this progression. First, RNA sequence and GSEA were analyzed in HCT116 transfected with miR-514b-3p, miR-514b-5p or control miRNAs. The enrichment plots of GSEA showed that metastasis associated gene signature and EMT pathways were enriched in cells with miR-514b-5p microRNA overexpression (Fig. 6a, b), and deficient in cells transfected with miR-514b-3p (Fig. 6c, d), but there is no difference in cells transfected with control microRNAs. These data demonstrated that miR-514b-3p and miR-514b-5p played vital roles in CRC EMT.

To further support our view, we tested the epithelial and mesenchymal markers level upon miR-514b-3p or miR-514b-5p overexpression. The results showed that the epithelial marker (E-cadherin and CLDN-1) increased in both mRNA and protein levels after ectopic miR-514b-3p expression, however, mesenchymal marker (fibronectin-1 and vimentin) were decreased (Fig. 6e, h). On the contrary, when cells were transfected with miR-514b-5p mimics, the expression level of E-cadherin, CLDN-1 were dramatically down-regulated, whereas mesenchymal markers were significantly up-regulated (Fig. 6e, h). These data confirmed our hypothesis that miR-514b-3p could suppress and miR-514b-5p might promote tumor metastasis via regulating EMT pathway (Fig. 6i).

## Discussion

Accumulating evidence have shown that miRNA could function as important modulators in tumorigenesis^21,22^, emerged as oncogenes or tumor suppressors, via different target genes. Detection of specific miRNAs in urine, blood, or tissue samples could serve as novel biomarkers and contributed to tumor diagnosis and clinical outcome prediction^23–26^.

In the current study, we analyzed the expression of miR-514b-3p and miR-514b-5p in 2 cohorts of CRC patients in Renji hospital. It is the first time to identify miR-514b-3p and miR-514b-5p expression in CRC. We found that miR-514b-3p was significantly downregulated in CRC compared with corresponding normal tissue, and decreased along with CRC progression. On the other hand, the expression of miR-514b-5p increased in CRC patients with advanced stage compared to patients with low clinical stage. These results implied that miR-514b-3p may act as a tumor suppressor gene, yet miR-514b-5p serves as an oncogene in CRC tumorigenesis, which might provide a possibility to predict CRC progression.

Tumor invasion and metastasis is the leading cause of cancer-related death worldwide. Numerus studies indicated that miRNA dysfunction was involved in tumor migration and invasion^27,28^. Here, we first evaluated the correlation between miR-514b-3p/5p expression and clinicopathological parameters in Renji cohort 1. Intriguingly, we found that the ratio of miR-514b-3p/5p expression was negatively correlated with lymph node invasion, AJCC stage and histological differentiation. Consistent with the finding, transfection of miR-514b-3p mimics damaged cell invasion ability and decreased metastatic CRC loci in the lung of nude mice. In contrast, overexpression of miR-514b-5p showed the opposite tendency. These data suggested that miR-514b-3p and miR-514b-5p involved in metastasis process in CRC development.

EMT is a morphogenic process which occurs during embryonic development, tissue repair, organ fibrosis and tumor invasion and metastasis^29,30^. During EMT, epithelial cells acquire fibroblast-like properties, exhibit reduced intercellular adhesion and show increased invasion ability. The activation of the epithelial-mesenchymal transition (EMT) program is generally considered as a major driver of tumor progression from initiation to metastasis. Given the fact that the role of EMT in promoting metastasis has recently been challenged, in particular concerning effects of the EMT transcription factors (EMT-TFs) Snail and Twist in pancreatic cancer^31^. Although this study showed that pancreatic cancer may metastasize without activating of EMT programs, the author’s conclusion can not be sustained by their data, because of the technical defect^32^. In addition, Krebs et al. showed that the EMT-TF Zeb1 is a key factor for the formation of precursor lesions, invasion and notably metastasis in the same pancreatic cancer model as well^33^. Furthermore, major studies remain support that EMT is crucial for tumor invasion and metastasis in majority type of cancers, including breast cancer, hepatocellular carcinoma, CRC, and our research model^34–38^. Our previous data demonstrated that miR-514b-3p and miR-514b-5p could regulate CRC invasion and metastasis, we next attempt to figure out whether this modulation was mediated by EMT. To confirm our assumption, we performed the GSEA analysis and detected the epithelial and mesenchymal markers in CRC cells transfected with miR-514b-3p or miR-514b-5p mimics. The results showed that miR-514b-3p and miR-514b-5p induced CRC invasion and metastasis was mediated by EMT. It was observed that patients with metastasis are prone to be more resistant to chemotherapy^39^. In addition, increasing evidences have demonstrated that chemoresistance is included in the EMT progression, which is the pivotal step in tumor invasion and metastasis^40^. Besides, Zheng et al further identified that EMT program induces chemoresistance in pancreatic cancer^31^. In this study, since miR-514 could influence EMT progress through target genes, we are supposed to further explore its function on chemoresistance. Interestingly, we found that miR-514b-3p, which suppresses EMT process, could undermine chemo-resistance in vitro or in vivo, in accordance with the fact that EMT induces stem cell properties, prevents apoptosis and contributes to immunosuppression^41^.

The specific mechanism of miR-514b-3p and miR-514b-5p involved in CRC is still unknown. To elucidate the underlying mechanism, we searched for three microRNA databases. E-cadherin and Claudin-1, translated from CDH1 and CLDN1, are well-known epithelial markers responsible for maintaining cell polarity and cell-cell adhesion^42,43^. Loss of them was considered as hallmarks of EMT process^44^. Our data showed that miR-514b-5p targets CDH1 and CLDN1, which was consistent with its function. In addition, we found that miR-514b-3p directly binds to 3′UTR of FZD4 and NTN1. It was documented that NTN1 could increase invasiveness and metastasis by promoting EMT process in hepatocellular and prostate carcinoma cancer cells^45,46^. FZD4 silencing induced E-cadherin expression, and thus restrained EMT initiation in human prostate cancer^47^. Consistent with previous studies, our results indicated that FZD4 and NTN1 were activated in CRC EMT process and could be blocked by miR-514b-3p overexpression.

There were still some deficiencies in our study. Further investigations were needed to reveal the underlying regulatory mechanisms which mediated the change in expression from precursor of miR-514 to mature miR-514b-3p and miR-514b-5p.

To sum up, the present study supplies the evidence of miR-514b-3p and miR-514b-5p involvement in CRC, for the first time. Functional studies demonstrated that miR-514b-3p and miR-514b-5p emerged to play distinct roles, both in vitro or in vivo, repressing or promoting tumor metastasis and chemoresistance, respectively. In line with previous studies, our discovery of the important role which miR-514 family plays in the CRC metastasis made it possible for miR514b-3p/5p used as a biomarker in tumor diagnosis and prognosis. Therefore, the detection and disruption of miR-514b-3p and miR-514b-5p could monitor and suppress CRC development, providing a new potential therapeutic strategy for human CRC.

## Electronic supplementary material

Supplementary Figure 1

Supplementary Table 1
