# Supplementary material for: The distinct role of strand-specific miR-514b-3p and miR-514b-5p in colorectal cancer metastasis
Source: Cell Death Dis. 2018 Jun 7;9(6):687. doi: 10.1038/s41419-018-0732-5 (PMC5992212; doi:10.1038/s41419-018-0732-5)
Supplement: Supplementary file 2 — Supplementary Table 1 [file 41419_2018_732_MOESM2_ESM.docx]

**Supplementary Table 1. The association between the ratio of miR-514b-3p/5p and clinicopathological features of CRC in Renji Cohort.**

| **Clinicopathological feature** | **Total**  **62** | **Ratio of miR-514b-3p/5p** | | **p Value**  **(x^2^ test)** |
| --- | --- | --- | --- | --- |
|  |  | **Low**  **31 (50%)** | **High**  **31 (50%)** |  |
| **Age (years)** |  |  |  |  |
| <65 | 38 | 22 (57.9%) | 16 (42.1%) | 0.118 |
| ≥65 | 24 | 9 (37.5%) | 15 (62.5%) |  |
| **Gender** |  |  |  |  |
| Male | 39 | 16 (41%) | 23 (59%) | 0.066 |
| Female | 23 | 15 (65.2%) | 8 (34.8%) |  |
| **Tumor size** |  |  |  |  |
| ≤4cm | 28 | 13 (46.4%) | 15 (53.6%) | 0.610 |
| >4cm | 34 | 18 (52.9%) | 16 (47.1%) |  |
| **Clinical stage (AJCC)** |  |  |  |  |
| Stage I / II | 30 | 10 (33.3%) | 20 (66.7%) | **0.011** |
| Stage III / IV | 32 | 21 (65.6%) | 11 (34.4%) |  |
| **T classification** |  |  |  |  |
| T1/T2/T3 | 30 | 13 (43.3%) | 17 (56.7%) | 0.309 |
| T4 | 32 | 18 (56.3%) | 14 (43.7%) |  |
| **Lymph node metastasis** |  |  |  |  |
| Absent | 35 | 13 (37.1%) | 22 (62.9%) | **0.021** |
| Present | 27 | 18 (66.7%) | 9 (33.3%) |  |
| **Distant metastasis** |  |  |  |  |
| Absent | 54 | 25 （46.3%） | 29 （53.7%）） | 0.130 |
| Present | 8 | 6 （75%）） | 2 （25%） |  |
| **Vascular invasion** |  |  |  |  |
| Absent | 53 | 26 (49.1%) | 27 (50.9%) | 0.718 |
| Present | 9 | 5 (55.6%) | 4 (44.4%) |  |
| **Histological differentiation** |  |  |  |  |
| Well/Moderate | 46 | 18 （39.1%） | 28 （60.9% ） | **0.004** |
| Poor | 16 | 13 (81.3%) | 3 (18.7%) |  |

The bold number represents *p*<0.05.
